# A Study of the Food Environment at Australian Family Day Care

**DOI:** 10.3390/nu11102395

**Published:** 2019-10-07

**Authors:** Ruth Wallace, Brennen Mills

**Affiliations:** School of Medical and Health Sciences, Edith Cowan University, Joondalup 6027, Australia

**Keywords:** family day care, food environment, healthy eating, children, Australia, nutrition knowledge

## Abstract

Overweight and obesity in childhood is a significant public health issue. Family day care (FDC) offers a setting where good eating habits can be nurtured in young children, yet often the food environment is unhealthy. This study aims to determine FDC educators’ knowledge and confidence about nutrition and the barriers and enablers to supporting healthy food environments. Australian FDC educators were recruited to a mixed methods study using self-administered e-surveys and qualitative in-depth interviews. The survey data (*n* = 188) revealed good knowledge about sugary foods, but poor knowledge of protein-rich foods. Nutrition knowledge was not associated with confidence to make nutrition recommendations. Interviews (*n* = 9) revealed parents’ dietary choices and educators’ personal beliefs as barriers to healthy food environments, although importantly, the FDC educator role was identified as being pivotal in supporting the health and wellbeing of children and their families. This study highlights that FDC-specific nutrition education and support is vital to ensure children at FDC and their families are exposed to healthy food environments. Research to investigate specific avenues for nutrition education promotion programs specifically designed to support the unique role played by FDC educators is needed, in order to support the long-term health and welfare of the next generation of Australians.

## 1. Introduction

Early childhood is a significant developmental period and early life experiences impact adult health. In 2014–2015, 20% of Australian children aged 2–4 years were overweight or affected by obesity, increasing their risk of breathing difficulties, hypertension, insulin resistance, and early markers of cardiovascular disease [1]. Overweight or obesity affected children have an increased risk of carrying this weight status into adulthood [2]. The healthcare costs associated with obesity in childhood are significant [3]. Early childhood education and care (ECEC) services are an important health promotion setting, as research suggests an association between children’s attendance and increased risk of overweight/obesity, especially if healthy eating and physical activity are not promoted [4].

Family day care (FDC) is a unique type of ECEC service, and although not as well-utilised as centre-based services, it is offered globally among high-income countries such as the US, UK, Canada, Australia, and European countries, such as Finland [5,6,7,8,9,10,11]. In Australia, family day care educators operate from their own homes to provide education and care for small groups of children aged 0–12 years, favoured by parents working irregular hours [7], who consider it a more convenient service [9]. FDC educators must be registered with a FDC ‘provider’, responsible for supporting, training and advising individual FDC educators [7]. More than 182,000 Australian children attend FDC for on average 30 hours/week [12], meaning this a setting where children can develop positive lifelong food habits [13] and be taught food literacy skills [14], influencing their future health and wellbeing [6]. However, research indicates healthy eating at FDC is often overlooked. For example, qualitative studies of FDC educators have found that food provision was not compliant with dietary guidelines, and that educators were role-modelling negative food behaviours and reported difficulties managing ‘fussy’ eaters [6,8]. Moreover, a lack of nutrition education training, inaccurate nutrition-related beliefs and perceptions, and poor child feeding practices were also present [5]. A common theme reported in the literature was the ineffectiveness of communication between FDC educators and parents about nutrition and food choices for children [5,6,8]. Furthermore, a US study found the food provided for children in FDC was high in sodium, but low in vegetables and whole grains [11]. 

There is a paucity of recent research about Australian FDC food environments. Between 1998–2002, a food and nutrition kit was distributed to all New South Wales FDCs, resulting in improved food quality, nutrition policies, and FDC educator nutrition knowledge [15]. In Victoria (2004–2008), the Romp and Chomp program aimed to support health-promoting FDC environments. Evaluation of this program demonstrated that the role-modelling of healthy behaviours by FDC educators improved, fewer unhealthy foods were provided, and increased numbers of FDC educators had completed nutrition training [16]. US research suggests that FDC educators tend to work in isolation, are not always supported by supervision or policy, and the quality of the care offered (including healthy eating) is typically self-determined [17]. Working in isolation and a lack of support is also reported by FDC Australia, the national peak body representing the sector [18]. 

All ECEC services, including FDC, are assessed and rated by their regulatory authority against the National Quality Standards. Healthy eating is assessed under Quality Area 2—Health and Safety, which states “healthy eating and physical activity are promoted and appropriate for each child” [19] and guides educators towards resources based on the Australian Dietary Guidelines. However, there are also other Australian nutrition education/support programs available for ECEC in general, such as Munch ‘n’ Move (NSW), Healthy Eating Advisory Service (VIC) and Supporting Nutrition for Australian Childcare (SNAC). It is unclear if these are considered appropriate by FDC educators or providers to serve their unique needs. 

Given the potentially long-lasting effects of overweight/obesity in childhood, and the numbers of children attending FDC, FDC educators are well-positioned to promote healthy food environments by providing nutritious food, nutrition education and positive role-modelling behaviours. However, research indicates that some aspects of this environment may be sub-optimal or absent [5,6,8,11], potentially impacting longer-term health outcomes for young children.

The aim of this study was to determine FDC educators’ nutrition knowledge, their confidence to make nutrition recommendations, and to identify barriers and enablers to providing a healthy food environment. These data will inform FDC training and resourcing needs around promoting healthy food environments for children. This research sought to answer the following research questions: What level of nutrition knowledge and confidence about healthy eating is found among FDC educators? What are the barriers and enablers to providing and promoting a healthy food environment in FDC?

## 2. Materials and Methods 

A mixed methods study design was utilised, including an online quantitative survey and in-depth, semi-structured, qualitative telephone interviews, to ensure a wide-reaching assessment of the Australian FDC food environment. Ethics clearance was obtained from the Human Research Ethics Committee at Edith Cowan University (#18295).

FDC educators were recruited as voluntary participants. The study was advertised Australia-wide via the Family Day Care Association e-newsletter and existing online networks (e.g., the SNAC website and Facebook page). A sampling frame of all FDC educators was obtained from www.mychild.gov.au (an Australian government online portal/database of all ECEC services) and invitations to participate were emailed to all FDC educators.

The quantitative data were collected anonymously via a self-completed survey that was administered via the Qualtrics survey collection and data analysis software (version ^XM^). The survey was adapted for the FDC setting from the SNACPlus study, a program that provided professional development and resources to embed in the nutrition curriculum and supportive food environments in early childhood education and care (ECEC) services [20]. The survey assessed nutrition knowledge and confidence to make nutrition recommendations. To ensure statistical rigour and generalisability of results, a power calculation was undertaken using G*Power v3.0.10 [21]. A sample of *n* = 176 participants would allow the detection of a small effect size for the main effects (effect size <30, *p* = 0.05, power = 80). Thus, we aimed to gather 180 responses to the online survey. The quantitative data were imported to SPSS v24.0 (IBM, NY, USA) for analysis. Simple descriptive statistics and *t*-tests were conducted to assess levels of nutrition knowledge and confidence.

Survey respondents who indicated their willingness to participate further provided qualitative interviews, conducted by telephone, which were audio-recorded with permission for ease of transcription. A question guide was developed based on the literature and research questions [22], and on previous research conducted by the first author in the ECEC setting [23]. This guide was piloted and revised among the research team and used to guide interviews whilst still allowing conversations to flow freely [24]. Saturation was reached at nine interviews as the participants were all FDC educators, a cohesive and homogenous sample group [25], and no new data were emerging to add richness or understanding [26]. Participant confidentiality was protected through pseudonyms. The qualitative data were transcribed verbatim by the lead author and imported into NVivo (Version12) for analysis. A manifest content analysis, as described by Bengtsson [27], allowed us to describe participant’s responses and categorise them according to the interview questions, defining what was visible and obvious. This analysis method allowed us to remain true to the participant’s original meanings and contexts they described.

## 3. Results

### 3.1. Quantitative Data

#### 3.1.1. Demographic Profile

There were 188 responses to the online survey, representing a 1.3% response rate. The majority of respondents were female (98%), aged >35 years (80%), and were Technical and Further Education (TAFE) or university qualified (77%). Work experience ranged from 0–3 years (37%) to >10 years (39%). This demographic profile is similar to that reported by Family Day Care Australia in 2019 [18]. We had a relatively good spread of responses across the different Australian states and territories, with the highest representation from New South Wales (34%), Queensland (28%), Western Australia (19%) and Victoria (13%). 

#### 3.1.2. Personal Nutrition Habits

Participants (*n* = 188) were asked about their own efforts to eat healthily. The majority (89%) reported they did so most of the time, or always. Similarly, participants reported their agreement/disagreement with the statement: “I do not need to make changes to my diet as it is healthy enough”. Fifty-seven percent strongly agreed or agreed, 17.5% were neutral, and 25.5% disagreed/strongly disagreed.

#### 3.1.3. Providing Food for the Children Attending their FDC

Sixty-one participants (32%) reported that all food was provided by parents/carers. Most of the remaining 127 participants (94%) reported they made conscious efforts to provide healthy food for children most of the time or always. 

Similarly, participants reported their agreement/disagreement with the statement: “I do not need to make changes to the food I provide for the children attending my Family Day Care as it is healthy enough”. Of those that did provide food, 65% reported they strongly agreed/agreed, although 21% were neutral and 14% disagreed/strongly disagreed.

A paired samples *t*-test suggested that participants were more likely to make conscious choices to provide healthy food for children attending their FDC than they were to make conscious choices to eat healthily themselves (*t*(126) = 7.753, *p* < 0.001). 

#### 3.1.4. FDC Educators Confidence to make Nutrition Recommendations to Parents

Participants rated their confidence on a scale of 1–10 (1 = not very confident; 10 = very confident). They were more confident making recommendations about limiting unhealthy snacks, sugary drinks and high sodium foods than they were about limiting solid fats and choosing low fat meats/poultry (Table 1).

#### 3.1.5. Confidence Recommending Serve Sizes for Children

Participants (*n* = 188) were asked the extent to which they agreed with the statement: “I am confident at estimating the recommended serve size of foods for children,” via a scale of 1–6 (1 = strongly agree; 6 = strongly disagree). About half strongly agreed/agreed (57%), 23% were neutral and 20% disagreed/strongly disagreed.

#### 3.1.6. General Nutrition Knowledge

Participants (*n* = 188) answered five questions about recommended core food servings and servesizes for children aged 2–3 years (Table 2).

- About half (55%) correctly identified 2–3 serves/day of vegetables as the recommended intake (28), whereas 39% incorrectly reported this as 4–5 serves/day.
- About one-quarter (27%) correctly reported low-fat dairy is recommended for children (28). Five participants (2.5%) thought dairy foods should be excluded.
- Forty-four participants (23%) correctly identified one serve of fruit/day as the recommendation (28), but 69% overstated this by suggesting 2–3 serves of fruit/day was required.
- The Australian Dietary Guidelines (ADG) state that one cup of tinned fruit is equal to one serving of fruit (28), which was correctly identified by 38% of participants, whereas 41% overestimated this measure, reporting one cup to be equivalent to 2–3 servings of fruit.
- About half (53%) correctly identified one cup of pasta to be equivalent to two servings of grain/cereal (28). A quarter (*n* = 48) underestimated one cup of pasta to be equivalent to one serving of grain/cereal.

**Figure 1:**
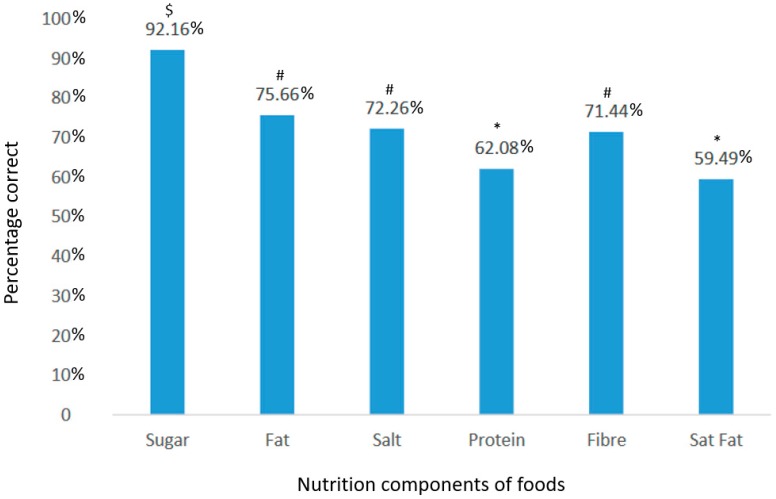
Percentage of correct responses for high vs. low nutrition content for specific foods (n = 188). ^$^ = statistically significantly greater compared to fat, salt, protein, fibre and saturated fat; ^#^ = statistically significantly less compared to sugar, and greater than protein and saturated fat; ^*^ = statistically significantly less compared to sugar, fat, salt, and fibre.

Participants were asked to identify if foods contained high or low sugar, fat, saturated fat, sodium, protein, and fibre content (Figure 1). Participants most accurately identified foods high or low in sugar but were least able to accurately identify foods high or low in protein and saturated fat (Figure 1).

#### 3.1.7. Nutrition Knowledge and its Impact on Confidence at Estimating Serving Size

The responses to the six nutrition category questions (i.e., sugar, fat, salt, protein, fibre and saturated fat) were summed to provide an overall indication of nutrition knowledge. Participants working as FDC educators for greater than six years achieved higher nutrition knowledge scores than those working as an FDC educators for less than six years (74% vs. 70% respectively, *p* = 0.034). The highest level of education completed (i.e., did not complete high school, Completed high school, Trade qualification, TAFE, University) had no bearing on nutrition knowledge (*p* = 0.317). 

Participants were asked if they agreed/disagreed with the statement: “I am confident at estimating recommended serve sizes of foods for children (1 = strongly agree; 7 = strongly disagree). From the 188 responses, 57% agreed/strongly agreed, 23% were neutral, and 20% disagreed/strongly disagreed.

A bivariate correlation analysis suggested there was no statistically significant linear relationship between confidence in predicting serving sizes of foods for children and their overall nutrition knowledge (*r* = −0.042, *p* = 0.567). A one-way ANOVA further suggested no statistically significant differences for knowledge score means between those that agreed (μ = 72% SD = 11.472), disagreed (μ = 70% SD = 15.353), or remained neutral (μ = 74% SD = 10.552) in terms of whether they were confident to estimate the serving sizes of foods for children (*p* = 0.375).

### 3.2. Qualitative Results

Nine female participants were interviewed, their FDC work experience ranging between one and 24 years. Participants described their reasons for working in FDC as a preference for working with small groups, thus facilitating close relationships with children and their families, and the family-style environment, allowing continuity of care. The most commonly reported disadvantage of FDC reported by participants was working in isolation from other educators or support mechanisms.

The following sections form a summary of data organised according to the questions from the question guide.

#### 3.2.1. Food Provided

Participants were asked what food they provided for the children attending their service and reported food provision on two distinct ends of a spectrum. For example, one participant reported providing three home-cooked hot meals/day, in contrast to another who provided no food at all (all food was provided by parents/carers).

The FDC educators who did provide food offered a wide range of meals/dishes, and in particular, described how they incorporated vegetables by offering carrot/zucchini noodles, vegetable pasta spirals; and adding finely chopped/grated vegetables to meals. 

One participant described the effort she spent preparing meals: “I do put a lot of effort into my food. I’m up at 6 [am] cooking a hot breakfast and hot lunches every day. I keep it [the food] as clean as possible, I tend not to do a lot of ‘snacky’ pre-packaged foods” (Cora). When asked the meaning of ‘clean’, she explained this meant unprocessed foods and creating meals from scratch.

#### 3.2.2. Providing a Healthy Food Environment

Participants were asked how they supported healthy eating at their service. Most reported they had vegetable/herb gardens, fruit trees and/or chickens, so children could harvest produce for meal preparation: “We have a veggie garden so quite often we’ll cook with what’s in the garden; carrot muffins, zucchini slice. We’ve got chickens so we might make something with the eggs” (Maureen).

Participants indicated that cooking with children was thought to encourage healthy food choices: “We do a lot of baking, the children and I feel like if I have them involved they can make choices about what they are eating and I’m beginning to see a pattern with them that they do make good choices” (Jane). 

Participants talked of role modelling positive food behaviours by sitting with children at mealtimes, eating the same foods: 

“If I have a good group of children, in terms of that they all get along really well, then I will sit down and eat my lunch with them and I will have the same as what they eat because I give them food that I’m happy to eat, and that’s always pretty healthy food”(Jane). Participants acknowledged that, at times, this practice was not always possible: “Yes, I do what I can [sitting with children at mealtimes], sometimes obviously it doesn’t happen, but I always intend to” (Noleen).

#### 3.2.3. Influences on Healthy Eating Environments

Participants were asked what made it difficult for them to provide a healthy food environment. They talked of the choices of foods by parents for their children, but participants own beliefs about food and healthy eating also emerged as a potential influence.

##### Parental Food Choices

Several participants noted that parents’ food choices for their children were not always healthy. For example, four participants recalled children regularly arriving at FDC with discretionary foods supplied by parents, e.g., lollies, chips, or fast foods. They recalled how difficult they found these situations to deal with, in light of their own efforts to promote healthy eating to the children: 

“If they’re coming to day care in the morning with a packet of chips, I can’t fight with that, it’s really difficult and I think it really takes the effort of everybody in that child’s life to make sure they are eating properly” (Jane).

One participant (Candy) suggested such parental food choices may be borne from low food literacy. She described a Fijian family, new to Australia and unfamiliar with the food environment, who were providing unhealthy foods for their child. This particular FDC educator posted information about healthy and discretionary foods on her Facebook page, and was able to influence the family’s food choices without appearing to criticise them directly.

Similarly, participants noted fussy eating as a barrier to a healthy food environment and described how they thought the contributing factors were related to parents’ dietary choices for their children. For example, one participant described caring for a child aged three years old, whose diet consisted of formula milk alone and who refused to eat solid foods, which the participant attributed to the parents lack of knowledge about age-appropriate feeding practices. Participants also noted how current food trends seemed to play a role in child fussy eating, describing how they thought parental requests for child’s diets to sometimes be based on popular or ‘fad’ diets, such as gluten-free diets.

##### FDC Educators Personal Beliefs

Some personal opinions expressed by participants did not align with the recommended dietary guidelines. For example, Jane expressed opinions that misaligned with the current evidence-based dietary guidelines, stating that she believed the ADG over-recommended cereals/grains and dairy foods. She claimed to have “done her own research”, subsequently adopting food practices such as limiting starches, using plant-based foods, and following the ‘I Quit Sugar’ diet [28].

Some participants’ expressed beliefs about providing discretionary foods that also conflicted with ADG recommendations. Discretionary foods, such as lollies, fast food, sweet biscuits and cakes typically have high sugar, fat, and sodium contents, and according to the ADG, should not be offered at ECEC [29]. One participant believed discretionary foods should be provided at FDC: “If you say children can only have healthy food, when they go out and make their own choices, they are ‘going to go crazy and just eat the poor choices’” (Mary). Another participant expressed that she was more concerned children were provided with enough food, i.e., that they do not go hungry, rather than being concerned about the nutritional content of this food. A participant who did not provide any food for the children in her care expressed her pragmatic approach to discretionary foods provided by parents: 

“I don’t judge—if someone sends in a lunchbox that is high in packaged foods and processed stuff, I think to myself well either a) they don’t know any different or b) they haven’t got time or c) that is all the child will eat. My challenge is getting those children who are used to that kind of food to try other things” (Noleen).

#### 3.2.4. Success Stories

Participants were asked about the successes they had experienced in embedding healthy eating practices at FDC. 

Several participants supported children to overcome fussy eating and adopt more balanced approaches to eating. For example, a participant (Annie) recounted how she cared for a boy (aged 2 years), who ate only pureed food, working with the child and his family over several months to slowly but successfully introduce solid foods to his diet. 

Participants recounted sharing resources with families, such as ideas on how to increase fruit and vegetable intake, and communicated success stories, such as when children ate vegetables they had previously refused. Resources and stories were reported to be mostly shared indirectly, for example, via service Facebook pages, with participants stating that they tended to avoid face-to-face conversations with parents about healthy eating. Polly recounted how children also relayed messages about food to their families: “Polly always eats healthy, there’s never any bad food at Polly’s house” or “no I can’t take that to day care because we’re not allowed to eat that food there”.

#### 3.2.5. Resources and Support 

Participants were asked what resources they already used to support healthy food environments at FDC, and what further support they might need. Six participants reported accessing ADG, the Australian Guide to Healthy Eating and other credible, evidence-based resources, whereas others stated that they simply searched the internet. Requests for additional support included access to general nutrition training, resources to share with parents and children, nutrition education resources, and meal preparation training. The participant who had implemented the “I Quit Sugar” program however, stated that she did not need any support: “I’m pretty good actually, that is probably the one area [healthy eating] that I feel most confident in” (Jane).

## 4. Discussion

This study aimed to establish the levels of nutrition knowledge and confidence about healthy eating found among FDC educators, and to understand the barriers and enablers to providing and promoting a health food environment at FDC.

### 4.1. Healthy Food Environment

More than two-thirds (67.5%) of participants provided food for children. Most of these participants (97%) stated they made conscious efforts to provide healthy food for children, and 65% stated they did not need to change the food provided as it was already healthy. However, survey data revealed that the general nutrition knowledge about the recommended serving sizes of core foods was less than encouraging (Table 2). Previous research findings have indicated the nutritional inadequacies of food provided in ECEC [6,8,11,30], highlighting disparities between educator confidence in their nutrition knowledge and actual practice, as reported in a recent Australian study [30]. 

During the interviews, participants described how they engaged children in healthy eating activities such as growing and harvesting vegetables and herbs, using these ingredients in cooking, and helping children make healthy food choices. Others talked of role-modelling positive food behaviours, such as sitting with children at mealtimes, but some acknowledged they often found this difficult to do. A NZ study of centre-based services reported 80% of educators were observed sitting with children whilst they ate [31]. It is possible that FDC educators are not fully aware of the contribution that sitting with children at meal-times can have towards positive role-modelling, and its greater overall contribution to providing a healthy food environment. Furthermore, the survey data suggest participants make greater conscious choices to provide healthy food for children attending FDC than making healthy choices for themselves, so they may be inadvertently role modelling poor food choices/behaviours to children.

Two barriers to healthy eating at FDC emerged, namely, the dietary choices of parents for their children and the participants own beliefs about food and nutrition. During the interviews, several participants lamented the food choices parents made for their children, believing these choices undermined the good habits they themselves were trying to embed, which has also been reported in another Australian study [23]. 

With regards to FDC educators’ own beliefs about food and nutrition, some participants expressed opinions about food provision that misaligned with the recommended nutrition guidelines. For example, Jane stated that she believed the ADG overprescribed cereals/grains and dairy foods, and having “done her own research” had subsequently implemented the ‘I Quit Sugar’ diet [28] at her FDC service. Similar stories have been described in another Australian study of ECEC food environments, where an educator recalled that a young child was prescribed a Paleolithic diet by her parents [23]. Relaying messages from popular diets without a basis in evidence, albeit unintentionally, is concerning, as educators have significant influence on the dietary intake of the child and in providing dietary information to their families [32]. 

### 4.2. Nutrition Knowledge and Confidence

Participants were asked to identify the correct number of serves for some core food groups for a child aged 2–3 years. Only 25–50% of participants correctly identified the correct number of serves for vegetables, fruit and cereals/grains, and only 27% correctly identified low-fat dairy as the recommendation for children aged >2 years. Similar results were reported in an Australian study, which also assessed nutrition knowledge among ECEC educators [20]. Two-thirds (68%) of participants thought the recommended fruit serves was 2–3 serves/day. Fruit is commonly overprovided in ECEC, and a ‘fruit fixation’ was reported in previous research that examined healthy eating environments in ECEC services [30]. Whilst fruit is a nutritious food, over-provision can displace other nutritionally important core foods. For example, almost all Australian children aged 2–3 years do not consume the recommended vegetable serves [33], and meat and dairy foods were reported to be under-provided at Western Australian ECEC services [34]. It is imperative that FDC educators understand the current, recommended, age-appropriate serves of core foods for children to support their optimal growth and development.

In terms of functional nutrition knowledge (i.e., understanding the key nutrients content in foods/meals), participants who had been FDC educators for >6 years scored higher than nutrition educators with <6 years of experience (74% vs. 70%, *p* = 0.034). These data are perhaps explained by results from an Australian study that found ECEC staff in senior roles, with more years of experience, were more likely to access nutrition information from professionally developed sources [30], such as ‘Eat for Health’ [29]. Participants had better knowledge about sugar, total fat, and the sodium content of foods/meals, than fibre, protein, or saturated fat content. Given the recent media attention about sugar-sweetened beverages [35], it is not surprising that the participants demonstrated good understanding of the sugar content of different foods/meals. It was concerning that knowledge about protein-rich foods/meals was lower, given the important role that protein plays in child growth and development [36]. Likewise, participants were more confident making recommendations to parents about limiting unhealthy snacks and drinks with added sugar and low sodium foods than they were about limiting solid fats and choosing low fat meats/poultry. Participants nutrition knowledge did not correlate with their confidence to make recommendations, suggesting that those with lower knowledge were just as confident to make recommendations as those with higher knowledge, again highlighting disparities between educator confidence in their nutrition knowledge and actual practices [30].

Participants demonstrated good knowledge of discretionary foods (i.e., foods high in added sugar, fat, and sodium) and corresponding confidence to discuss these foods with parents. However, some believed discretionary foods should be offered occasionally at FDC to protect children from overconsumption in other settings, contrary to dietary guidelines [29]. Others considered it more important the child had eaten enough food, rather than having concerns about the nutritional quality of the food. Similar opinions were reported among educators in other studies [37] but are problematic from a health promotion perspective, as children aged 2–3 years reportedly consume 30% of their daily energy needs from discretionary foods [38], potentially diluting the nutritional quality of their diets [37].

### 4.3. The Pivotal Role of FDC Educators

Participants recalled stories of how they promoted healthy eating while educating and caring for children, demonstrating the pivotal role of FDC educators in the life of a child and their family. For example, a FDC educator reported how she cared for a boy (aged 2 years) who would accept only pureed foods. Working with the family, she slowly introducing solid foods to his diet, successfully demonstrating the importance of repeated exposures to new foods [39]. Another FDC educator worked closely with a migrant parent to increase food literacy skills. Participants felt it important to communicate with parents about their children’s food and nutrition but expressed frustration about the discord between their own dietary objectives and those of the children’s parents [6]. UK research described that often parents feel more comfortable approaching their childcare provider than they would any other professional [6], and this is mirrored in the recent Family Day Care Australia report [18], that notes FDC educators enjoy quality relationships with children and their families. It is, therefore, essential that FDC educators have access to evidence-based nutrition training and resources to provide healthy food environments that support optimal health for the child and their family.

### 4.4. Strengths and Limitations

The mixed methods design of this research increased methodological rigour [40] and provided opportunities to present a holistic picture of the FDC food environment. The self-selected nature of the sample, as well as the relatively small response rate in consideration of our greater sampling frame, may be a limitation, as it is possible those with a predisposed interest in healthy food and eating may have been more inclined to participate, potentially questioning the ability of our results to be generalizable to the greater population of Australian FDC educators. It is unclear the extent to which self-selection bias may have also impacted on qualitative findings. Although, were this the case, it is likely the nutrition knowledge reported from this study may actually be greater than that of the true population, only further demonstrating the need for further intervention in this space. Conversely, it is also plausible some may have chosen to participate as they maintained a self-perception that their nutrition knowledge was lacking and were hoping to learn from participation in the research. The extent to which the potential for these self-selection biases to impact on the generalisability of study results is unclear but should be considered.

## 5. Conclusions

Establishing healthy food habits in young children is essential if they are to meet their full potential. This study revealed the fact that nutrition knowledge among FDC educators varies, yet there were some key nutrients for which knowledge was low. This is concerning, as the majority of FDC educators surveyed did not believe they needed to adapt the food they offered children, presuming it was healthy enough. Equally concerning, participants with low nutrition knowledge were just as likely to make nutrition recommendations to parents as those with better nutrition knowledge. However, the significant role of the FDC educators as a central support mechanism for children and their families emerged. Given this important role, FDC-specific nutrition education and support is vital to ensure children at FDC are exposed to healthy food environments, and that this support extends to the families of the children attending FDC. Further research is needed to investigate specific avenues for nutrition education promotion programs specifically designed to support the unique role played by FDC educators. The evaluation of such programs would ideally involve direct observations of nutrition-related behaviours in the FDC environment. It is clear this cohort plays an inherent role in forming relationships with food and healthy eating amongst young children and their families. Working to ensure FDC educators are providing food and nutrition advice in line with Australian Dietary Guidelines could have a dramatic and measurable impact, increasing improved healthy eating behaviours amongst our next generation.

## Figures and Tables

**Table 1:** Family day care (FDC) educators’ confidence of making nutritional recommendations to parents (n = 188).

No.	Question	Mean (/10)	SD
1	Limiting unhealthy snacks, e.g., lollies, biscuits, cakes, chips, fast foods	8.76 ^#^	3.168
2	Limiting sugary drinks	9.02 ^#^	3.301
3	Eating foods with low sodium content	8.22 ^^^	3.316
4	Eating few solid fats and foods that contain these, e.g., butter, margarine, shortening, lard.	7.66 ^&^	3.464
5	Eating low-fat meats or poultry	7.62 ^&^	3.563

*^#^ =* Statistically significantly greater compared to items 3, 4, and 5. (α = 0.05); ^^^ = statistically significantly less compared to items 1 and 2, and greater than 4 and 5. (α = 0.05); ^&^ = statistically significantly less compared to items 1, 2 and 3. (α = 0.05).

**Table 2:** Number of participants answering food group nutrition questions (for children aged 2–3 years, per the Australian Dietary Guidelines (ADG)) correctly (*n* = 188).

No.	Question	N	%
1	How many serves of vegetables/day are recommended?	104	55
2	What types of dairy foods are recommended?	51	27
3	How many serves of fruit/day are recommended?	44	23
4	1 cup of pasta provides how many serves from the “grains/cereals” group?	99	53
5	1 cup of tinned fruit provides how many serves from the "fruit" group?	72	38
